# Psychological Well-Being of Adult Psoriasis Patients: A Narrative Review

**DOI:** 10.7759/cureus.37702

**Published:** 2023-04-17

**Authors:** Ankita Hepat, Swarupa Chakole, Asmita Rannaware

**Affiliations:** 1 School of Epidemiology and Public Health, Jawaharlal Nehru Medical College, Datta Meghe Institute of Higher Education and Research, Wardha, IND

**Keywords:** young adult, substance abuse, stress, depression and anxiety, psychological comorbidities, psychological well-being, psoriasis

## Abstract

Psoriasis is a chronic systemic inflammatory condition, and psychiatric comorbidities are common in this condition. It is a non-communicable, autoimmune, and incurable disease. Psoriasis causes an adverse effect and is connected with various psychological symptoms like social isolation, guilt, and embarrassment of a patient. They decrease self-esteem because of depression, anxiety, stress, and substance abuse adults. The prevalence rate of adults is gradually increasing. This study uses various scales to evaluate the level of psoriasis. This study aims to evaluate the level of depression, anxiety, stress, and substance abuse among adult psoriasis patients and to identify the factors affecting psoriasis patients. A detailed search was carried out using essential databases such as PubMed, Google Scholar, and the World Health Organization (WHO) database to search for articles elucidating the same. In total articles, 36 out of 160 are selected. All studies found that psoriasis is at a positive level in that the level of depression and anxiety is moderate to severe, the level of stress is moderate, the level of alcohol abuse is higher, and the level of smoking consumption is ever-increasing. A severe skin condition that impacts the quality of life and psychological health. It has become a public health issue. All the selected articles assessed patients who were highly affected by depression, anxiety, stress, and abuse. They also assessed the various comorbidities related to psoriasis.

## Introduction and background

Psoriasis is a typical incurable, comprehensive inflammatory phase that impacts around 2% of the world's population [1]. According to the World Health Organization (WHO), the adult age group is above 19 [2]. Psoriasis prevalence ranges from an adult is 0.51% to 11.43% globally [2]. According to a recent epidemiological study, psoriasis affects about 125 million individuals worldwide and is gradually becoming more common [2]. And also, the prevalence rate of psoriasis is different among various populations, ranging from 0% to 11.8%. In India, it varies from 0.44% to 2.8% [3]. According to the World Health Organization (WHO), psoriasis is a group of non-communicable diseases (NCDs) [4]. Psoriasis is incurable, non-contagious, leaves unpleasant scars all over the body, and is an untreatable disease for which there is no cure and leaves harmful effects on a patient’s quality of life (QoL). It can arise in any age group, most frequently in the adult age. The exact incidence of psoriasis in the nation varies from 0.09% to 11.4%, and the characteristics of psoriasis are a significant global issue [5].

Psoriasis impacts the skin and nails [6]. The most typical signs and symptoms associated with psoriasis are scaling of the skin at 92%, excessive itching at 72%, erythema and redness at 69%, tiredness and lethargy at 27%, inflammation at 23%, burning at 20%, and bleeding at 20% of patients; it is associated with several comorbidities [5]. It brings about several mood swings in young adults. The global burden of disease study found that 5.6 million persons of all age groups had disability-adjusted life years (DALYs) related to psoriasis in 2016 [4]. Another term for psoriasis is "systemic disease having multiple comorbidities,” i.e., cardiovascular disease (CVD), psoriatic arthritis (PSA), and mental disorders [7]. Psoriasis is the most common chronic skin disorder, negatively affecting psychological interactions and health. Hence, psoriasis may conduct in reducing the self-esteem of the patients. Psoriasis patients usually experience an increased level of anger [8]. Psoriasis causes magnificent physical, emotional, and social burdens [5], leading to higher rates of depression, anxiety, stress, and substance abuse more frequently [9]. Psoriasis is thought to affect 3% of people worldwide, though important regional and demographic differences have been documented [10]. The main motive of the study is to identify whether psychological intervention is an effective treatment to relieve both psoriasis and psychological well-being [11]. The aim is to evaluate the quality of life, severity of stress and assess depression, anxiety, stress and substance abuse of patients with psoriasis [9].

The quality of life, in the conditions of well-being and disease, is a multidimensional phase that selects good health and satisfaction on how health is impacted, such as the state of one's emotions, physical functioning, and social well-being [12]. It must be emphasized that sufferers of chronic dermatoses like psoriasis, who progressively develop more skin lesions, or whose dermatitis lesions do not even react to medications or their site is critical for the patient when compounded with mental health issues, as well as a social relationship, show a lack of personal growth, purpose in life, autonomy, self-acceptance, positive relations with others and environmental mastery [13]. Acceptance of illness is defined as the lack of unfavorable responses and reactions related to the condition [14].

## Review

Methodology

**Figure 1:**
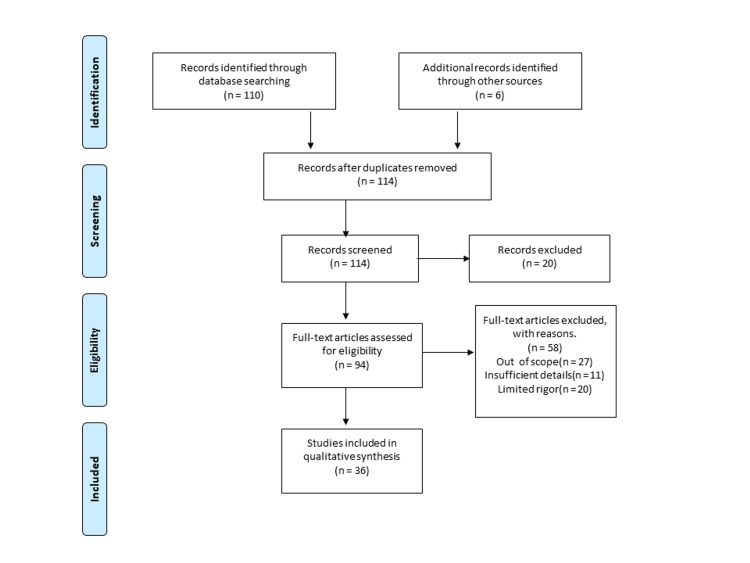
Inclusion and exclusion criteria of the study.

This paper discusses the psychological well-being of adult psoriasis patients. We search Medline via PubMed, Google Scholar, and databases like WHO. The search strategy for PubMed was tailored to individual databases and was as (mental health (Title/Abstract)) AND (psoriasis (Title/Abstract)). Furthermore, we screened the referenced list of potentially relevant studies to seek additional studies. The language of the study is English, and articles in other languages were excluded. Articles ranging from 2000 to 2022 were used. Additional filters, such as free full text, are applied to all research. For more detail, we used various keywords like “psychological well-being,” “psoriasis," “depression and anxiety,” “stress,” “substance abuse,” “young adult,” and “psychological comorbidities.” Figure 1 shows inclusion and exclusion criteria.

Psychiatric comorbidities in psoriasis

Psoriasis is commonly associated with mental disorders, especially depression and anxiety [7]. Psoriasis is a lifelong relapsing-remitting illness that negatively impacts people's quality of life (QoL). Stress, substance abuse, anxiety, and depression are among the psychosocial comorbidities that are closely linked to psoriasis. The all-party parliamentary group on skin (APPGS) recently published a study that highlights the severity of the mental health burden skin diseases pose in the United Kingdom (UK). In rheumatic disorders mostly and in psoriatic conditions in specific, systemic inflammation results in large no of insulin resistance, endothelial cell dysfunctions, atherosclerosis, development of cardiovascular diseases (CVD) [15]. Increased risk of several comorbidities, such as cardiovascular and other non-communicable disorders, is also related to psoriasis. Psychiatric disorders are among the comorbidities connected with psoriasis patients, with 40% to 90% higher psychosocial comorbidities than the general population [16]. Psoriasis, also called a chronic disorder, occurs by various comorbidities. Firstly, it is related to psoriatic arthritis (PSA), which influences around 5% to 40% of patients with psoriasis disease and regularly guides them to physical disability. Assessing the personal sign of skin disease, comorbidities, and frequently time-saving or cost-effective medical care has more effect on patients with psoriasis diseases' low standard of living [9]. Recognition and management of comorbidities (such as psoriatic arthritis, psychological, cardiovascular, and hepatic disorders) is an essential part of holistic care for individuals with psoriasis [4].

Discussion

People are cautious about their body image and appearance [13]. Hence, chronic conditions like psoriasis, identified by a higher frequency and apparent skin discoloration, are a specific burden for patients regarding poor body image and social perception. Stress and other psychological and social issues result from being aware of one's imperfect appearance, which is not accepted by society [13]. Psoriasis is evaluated by the region of skin affected (body surface area (BSA)) and the severity of the scaling, induration, and erythema. In secondary care, confirm results similar to the psoriasis area severity index (PASI) and physician global assessment scale (PGAS) along with patient-reported outcomes that have been measured, like the dermatology life quality index (DLQI) [4,17]. The first study of psoriasis to describe the relationship between depression and anxiety as measured by reliable screening tools [18]. The main findings of our study were that individuals with psoriasis had significantly higher levels of stress, anxiety, and depression when compared to healthy controls. Second, there was no significant connecting disorder period, and psoriasis area severity index (PASI) scores calculated disease severity and levels of depression, anxiety, and stress. Third, even though the relation between depression and anxiety stages and disease severity in a patient with psoriasis has often been researched, few studies have examined the association between community anxiety and becoming aware of stress and the acuteness of the disease. To the best of our understanding, this research is the first to report the relation between psoriasis patients’ susceptibility to anxiety and the severity of their condition [19].

Depression and Anxiety

Depression is a chronic psychological illness. Around the world, 5% of adults suffer from these conditions [20]. It is defined as continuous sadness and a lack of interest or happiness before pleasurable or satisfying activities [20]. World Health Organization (WHO) states that a feeling of tension, worried thoughts, and physical changes like higher blood pressure are all characteristics of anxiety [20]. A study explains how all their subjects are evaluated using the patient health questionnaire (PHQ-9) for depression and anxiety, perceived stress, and quality of life [6]. Generalized Anxiety Disorder Scale (GAD-7), Perceived Stress Scale (PSS), World Health Organization Quality of Life (WHOQOL-Bref) [21], and Psoriasis Area and Severity Index (PASI) are the most commonly used standard scales [22]. The objective of the study, which measured the generality of depressive and anxiety signs with more than a combined assessment based on a meta-analysis of depression signs of psoriasis patients, detailed how depression and anxiety are everywhere in patients with psoriasis. It assessed patient questionnaires and reported a prevalence of 28% [6], evaluated the level of cases with moderate-to-severe psoriasis with depression, anxiety, or both, and that the patients will probably experience physical or mental problems in their lives. Early diagnosis of the psychological problem is essential for enhancing clinical outcomes and well-being-related quality of life. Some investigations have shown that depression and anxiety also belong to common inflammatory disorders [23]. The study also explained how patients with psoriasis are 1.5 times more prone to depressive signs when compared with the control group, and more than 20% of psoriasis patients experience depression and anxiety at some point in their lifetime when afflicted with psoriasis [6]. Akay et al. assessed the degree of depression in psoriasis patients and established that depression's severity level is significantly higher in psoriasis cases than in the control group [19]. Table 1 depicts the outcome percentage of depression and anxiety in psoriasis patients.

**Table 1:** Outcomes percentage of depression and anxiety in psoriasis patients.

Name of country	Used scale	Percentages of symptoms	Result
Lakshmy et al. India patients [21]	World Health Organization Quality of Life (WHOQOL-Bref), Perceived Stress Scale (PSS), Patient Health Questionnaire (PHQ9), Generalized Anxiety Disorder Scale (GAD-7)	Depressive sign: 78.9% and anxiety sign: 76.7%	This study establishes the result of severe anxiety and moderate-severe depression.
Tian et al. Chinese patients [24]	Patient Health Questionnaire (PHQ-9), Generalized Anxiety Disorder Scale (GAD-7), and Psoriasis Area and Severity Index (PASI)	Depressive sign: 13.9% and anxiety sign: 10.6%	They were established to be helpful factors for developing moderate-to-severe depression or anxiety features, although a prolonged period and late-onset age performed a safety part.
Akay et al. Turkey patient [25]	Beck Depression Inventory (BDI), Beck Anxiety Inventory (BAI)	Depressive sign: 58%	This study found that depression and anxiety are assessed severity scores high in psoriasis.

Stress

Stress can be classified as the variety of changes that cause physical, emotional, or social strain [26]. Your body responds to something that requires consideration or action by causing stress [26]. Many psoriasis patients experience a particular problem with illness-related stress (IRS). Illness-related stress (IRS) mostly arises from the expectation of others’ responses to psoriasis. The increasing number of various scales, including health-related quality of life (HRQOL), considers and describes the present approach to psoriasis [27]. One of the factors that could contribute to the emergence and progression of this illness is stress. There is considered to be a very strong connection between the two, and no disorder or other factors are known to cause this kind of interaction. It consists of seven complaints about being sensitive, anxious, overreacting to wind down, and finding it challenging to rest [6].

Leovigildo et al. conducted a stress evaluation using Lipp's stress symptoms inventory (LSSI) for an adult to evaluate the stress level. According to the three stress stages, the questionnaire associates and characterizes physical and emotional indicators such as alarm, resistance, and tiredness [28]. The stress subscale of the Depression Anxiety Stress Scale-21 (DASS-21) quantitatively calculates stress [21]. According to the outcomes, it was found that 85% of the patients had a clinical diagnosis of stress: 48% were in the opposition stage, and 37% were in the tiredness stage [28].

Substance abuse

Alcohol

Alcohol is a psychoactive drug with addictive qualities that has been used globally across various religions for the nation. The dangerous use of alcohol can cause a chronic disease burden and has a substantial economic and psychological impact [29]. Excessive consumption of alcohol must be discouraged due to the cumulative effects of alcohol on psoriasis patients at higher risk of liver damage [30]. Patients with psoriasis used more alcohol than familiar people. This study used questionnaires on alcohol dependence: cut, annoyed, guilty, and eye (CAGE) and self-administered alcohol screening test (SAAST) questionnaires to measure the alcohol patient with psoriasis [31]. People suffering from psoriasis have an increased alcohol-related death risk. It was established that psoriasis patients suffering from a 60% increased probability of death from alcohol-associated causes are different from others of similar age and sex in common people [32]. It concluded that alcohol intake will increase the level of psoriasis, but not that it is a risk factor for developing psoriasis [31].

Smoking

We classified present cigarette consumers as those consumers who continued to smoke at the end of the examination and previous cigarette consumers as these consumers who had abstained from smoking for and above at the end of three months of the examination [33]. Cigarette smoking has been considered a predisposing factor for developing psoriasis. Current and former smokers are more susceptible to developing psoriasis than non-smokers. The study performed aimed at discovering the relation between the severity of the illness and the frequency of habits and cigarette use among psoriasis patients [34]. Harmful behaviors like smoking and drinking are common among psoriasis patients, and this is influenced by the condition's chronicity and incurability of psoriasis [34]. Tobacco causes the death of more than eight million populations in every year. Overall, 1.2 million people die and are impacted by non-consumers life exposed to second-hand smoke, although more than seven million people regularly smoke. More than 80% of the 1.3 billion people in the world are cigarette users and live in low-income and middle-income countries [35]. Psoriasis disorder severity was evaluated by the psoriasis area and severity index (PASI) [33]. In a study by Salihbegovic, and he saw a higher occurrence of smoking cigarettes in psoriasis patients and more frequent smoking cigarettes in psoriasis patients than in controls [34].

Quality of Life

Quality of life (QOL) is a method for patients to recognize and respond to their health issues and other aspects of their life. According to this point of view, all quality of life (QOL) includes factors such as family and friends, like physical, emotional, functional, and intellectual well-being. In spite of various concepts [36], these factors influence the patient's assessment of how their condition affects their quality of life and the effects of the condition on their personal and professional objectives [12]. Additional to assessing the clinical symptoms, a health-related quality of life (HRQOL) is an essential component for assessing the development of the disorder and its result on the patients' health and well-being. The dermatology life quality index (DLQI) is also used [5].

Factors Affecting Psychological Well-Being in Psoriasis

**Figure 2:**
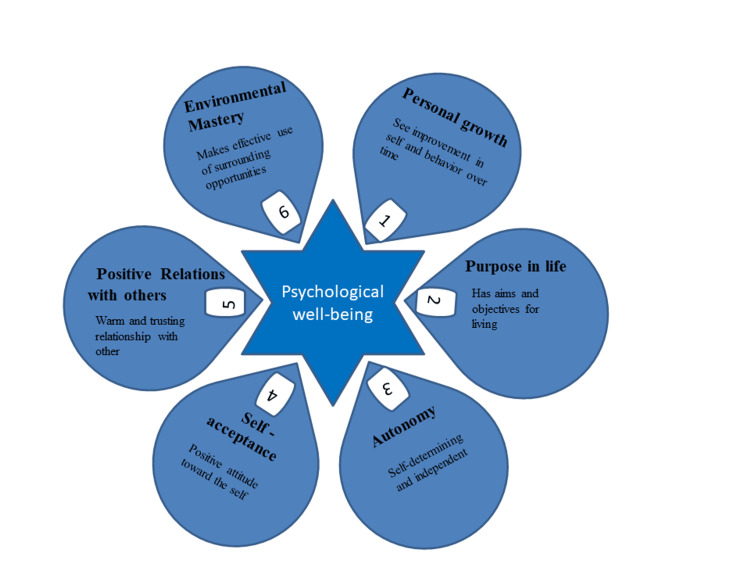
Factors affecting psychological well-being in psoriasis. This image is the author's own creation.

The various psychological problems, including social relationships, show a lack of individual growth, purpose in life, autonomy, self-acceptance, positive relation with others, and environmental mastery in people who suffer from psoriasis. Figure 2 depicts factors affecting psychological well-being in psoriasis.

## Conclusions

In conclusion, psoriasis is a chronic, incurable, and comprehensive inflammatory condition that affects a significant proportion of the world's population. It impacts the skin and nails and is associated with several comorbidities, such as cardiovascular disease, psoriatic arthritis, and mental disorders. Psoriasis negatively affects psychological interactions and health, leading to depression, anxiety, stress, and substance abuse. The quality of life of psoriasis patients is multidimensional and includes emotional, physical, and social well-being. Hence, this review helps to improve psoriasis sufferers' mental health using psychotherapy, prompt and efficient inflammation treatment, and meditation. Psoriasis is commonly associated with mental disorders, especially depression, and anxiety. Psoriasis is a significant global issue that requires appropriate management and care to alleviate its impact on patients' quality of life. There are various methods to find out how deeply psoriasis affects people, like the perceived stress scale (PSS), the World Health Organization's (WHO) Quality of life (WHOQOL-Bref), Patient Health Questionnaires (PHQ-9), and the Generalized Anxiety Disorder scale (GAD-7). The findings may indicate the level of psoriasis and its psychological outcome and help to determine the rate of depression, anxiety, stress, and substance abuse. Patients’ evaluation of the disease because it impacts their quality of life on a social, psychological, and physical level is also important, and acknowledging that there will be several hardships associated with being a person who has psoriasis is vital because that way, they will be able to make life decisions and carry themselves in the society with ease, knowing they have a condition that is unique and sometimes in life they might be subjected to difficulties that might challenge them at different phases in their lives. They might experience a lack of individual growth, purpose in life, autonomy, self-acceptance, positive relations with others and environmental mastery; nevertheless, we must never lose hope for a cure necessitating extensive research and approaches to diminish the various effects of psoriasis.
